# Predicting factors for acute encephalopathy in febrile seizure children with SARS-CoV-2 omicron variant: a retrospective study

**DOI:** 10.1186/s12887-024-04699-x

**Published:** 2024-03-25

**Authors:** Ching-Min Tang, Cheng-Yen Kuo, Chen-Wei Yen, Jainn-Jim Lin, Yu-Chia Hsieh, Shao-Hsuan Hsia, Oi-Wa Chan, En-Pei Lee, Po-Cheng Hung, Huei-Shyong Wang, Kuang-Lin Lin, Cheng-Hsun Chiu

**Affiliations:** 1 Division of Pediatric Neurology, Chang Gung Children’s Hospital and Chang Gung Memorial Hospital, Kwei-Shan, 5 Fu-Shin Street, Taoyuan, 333 Taiwan; 2 School of Medicine, College of Medicine, Chang Gung University, Taoyuan, Taiwan; 3 Division of Pediatric General Medicine, Department of Pediatrics, Chang Gung Memorial Hospital, Taoyuan, Taiwan; 4 Division of Pediatric Critical Care and Pediatric Neurocritical Care Center, Chang Gung Children’s Hospital and Chang Gung Memorial Hospital, Taoyuan, Taiwan; 5 Division of Infectious Diseases, Department of Pediatrics, Chang Gung Memorial Hospital, Taoyuan, Taiwan; 6 Division of Pediatric Infectious Diseases, Molecular Infectious Disease Research Center, Chang Gung Memorial Hospital, Chang Gung University College of Medicine, Taoyuan, Taiwan

**Keywords:** Pediatric, SARS-CoV-2, COVID-19, Febrile seizures, Encephalopathy, Predictive factor

## Abstract

**Background:**

SARS-CoV-2 posed a threat to children during the early phase of Omicron wave because many patients presented with febrile seizures. The study aimed to investigate predicting factors for acute encephalopathy of children infected by SARS-CoV-2 Omicron variant presenting with febrile seizures.

**Methods:**

The retrospective study analyzed data from pediatric patients who visited the emergency department of Chang Gung Memorial Hospital in Taiwan between April and July 2022. We specifically focused on children with COVID-19 who presented with febrile seizures, collecting demographic, clinical, and laboratory data at the pediatric emergency department, as well as final discharge diagnoses. Subsequently, we conducted a comparative analysis of the clinical and laboratory characteristics between patients diagnosed with acute encephalopathy and those with other causes of febrile seizures.

**Results:**

Overall, 10,878 children were included, of which 260 patients presented with febrile seizures. Among them, 116 individuals tested positive for SARS-CoV-2 and of them, 14 subsequently developed acute encephalopathy (12%). Those with acute encephalopathy displayed distinctive features, including older age (5.1 vs. 2.6 years old), longer fever duration preceding the first seizure (1.6 vs. 0.9 days), cluster seizure (50% vs. 16.7%), status epilepticus (50% vs. 13.7%) and occurrences of bradycardia (26.8% vs. 0%) and hypotension (14.3% vs. 0%) in the encephalopathy group. Besides, the laboratory findings in the encephalopathy group are characterized by hyperglycemia (mean (95% CI) 146 mg/dL (95% CI 109–157) vs. 108 mg/dL (95% CI 103–114) and metabolic acidosis (mean (95% CI) pH 7.29(95% CI 7.22–7.36) vs. 7.39 (95%CI 7.37–7.41)).

**Conclusions:**

In pediatric patients with COVID-19-related febrile seizures, the occurrence of seizures beyond the first day of fever, bradycardia, clustered seizures, status epilepticus, hyperglycemia, and metabolic acidosis should raise concerns about acute encephalitis/encephalopathy. However, the highest body temperature and the severity of leukocytosis or C-reactive protein levels were not associated with poor outcomes.

**Supplementary Information:**

The online version contains supplementary material available at 10.1186/s12887-024-04699-x.

## Introduction

While SARS-CoV-2 primarily targets the respiratory system, neurological manifestations are observed in 80% of hospitalized patients. Among these, acute encephalopathy constitutes 49% of the neurological symptoms and signs [1]. Various encephalopathy/encephalitis may arise, including infection-triggered encephalopathy syndrome (ITES), such as acute necrotizing encephalopathy (ANE), acute encephalopathy with biphasic seizures and late reduced diffusion (AESD), encephalopathy with acute fulminant cerebral edema, mild encephalopathy with reversible splenial lesions (MERS), hemorrhagic shock and encephalopathy syndrome (HSES), as well as acute encephalopathy of unknown cause or unclassified, typically occurring during acute febrile illness [2]. Additionally, demyelination disorders like acute disseminated encephalomyelitis (ADEM) and acute hemorrhagic leukoencephalitis (AHLE) may manifest during both acute and post-infection periods [3–5]. While uncommon, approximately 19 cases of autoimmune encephalitis (AE) have been reported in association with SARS-CoV-2 infection [6]. These cases typically present as acute or subacute clinical courses and encompass conditions such as limbic encephalitis, anti-NMDAR encephalitis, new-onset refractory status epilepticus (NORSE), and instances with an unidentified type of AE. Despite the various types of encephalitis associated with SARS-CoV-2 infection, the virus is rarely detected in the central nervous system [7], suggesting the diverse spectrum of encephalopathy/ encephalitis and the complexity of the immune response to the virus.

The classification of infection-triggered encephalopathy syndrome typically occurs in the late stage of the disease, following the acquisition of cerebrospinal fluid, brain imaging, and specific antibodies. However, timely intervention is crucial for favorable outcomes [8]. Understanding how to predict which children with febrile seizures will develop acute encephalopathy is paramount. Previous studies in Japan have identified factors such as prolonged or refractory febrile seizures that increase the risk of acute encephalitis [9].

The primary objective of this study is to identify more potential predictive factors for acute encephalopathy in children with febrile seizures and SARS-CoV-2 infection. The findings might significantly impact both clinical management and public health strategies. By recognizing risk factors associated with developing encephalopathy, healthcare providers can promptly identify and manage encephalopathy, potentially improving outcomes and reducing morbidity and mortality. Public health efforts can also benefit by incorporating these risk factors into surveillance and prevention strategies, helping to mitigate the risk and burden of severe neurological complications associated with pediatric COVID-19 cases. Overall, the study provides valuable insights for guiding clinical decision-making and public health policies related to febrile seizure in pediatric COVID-19 patients.

## Subjects and methods

### Study design and participants

**Fig. 1:**
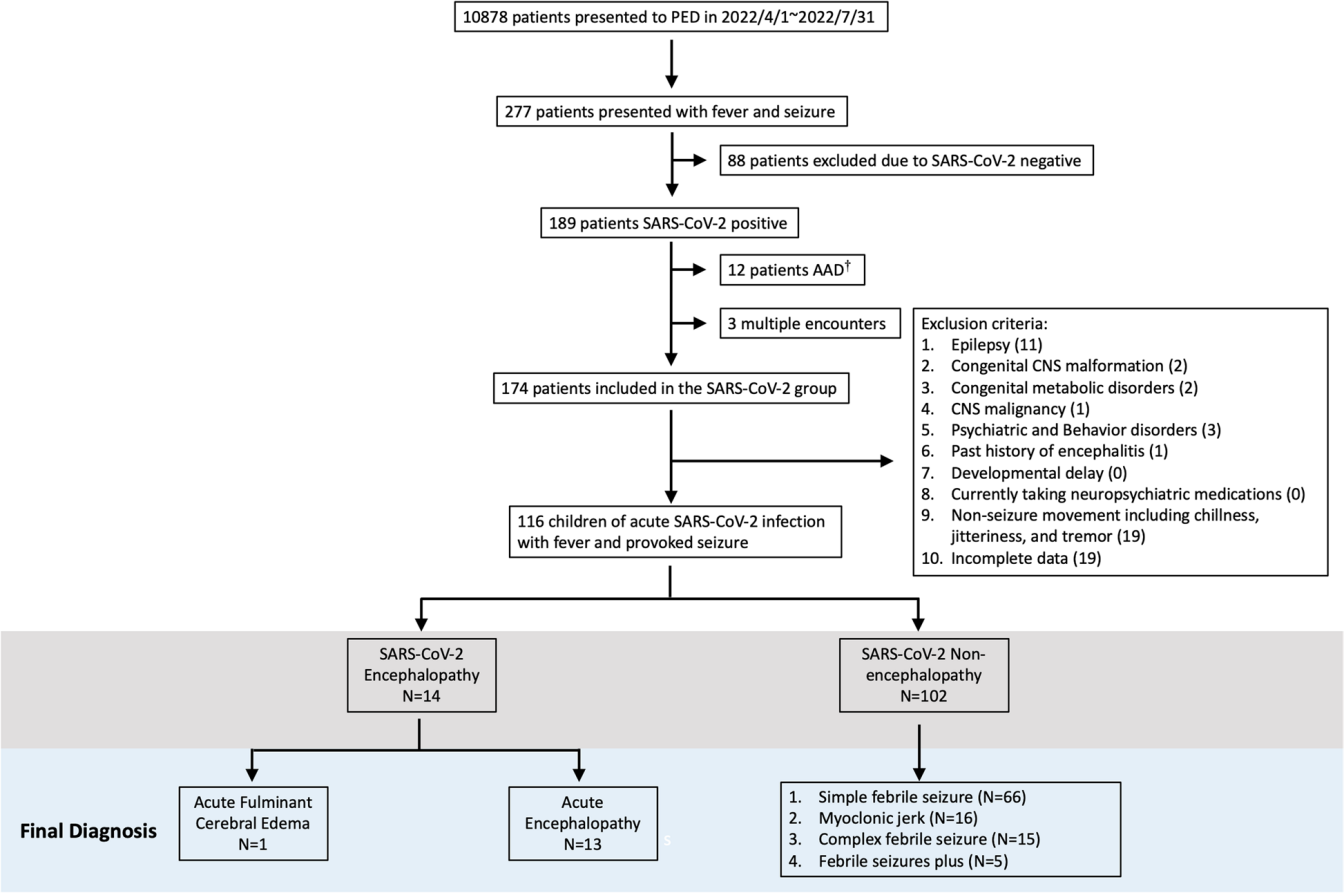
Flow chart of the study. The overall number of the entire cohort of children is given first. Patients were included and excluded according to the selection criteria. The gray background represents the tentative diagnosis at the pediatric emergency department, and the blue background represents the final diagnosis of the population. †AAD, Against Advice Discharge. Numbers in the round brackets in the box of exclusion criteria represent patient numbers

We performed a retrospective chart review of patients who visited the PED of Chang Gung Memory Hospital, Linkou Branch, a referral medical center in northern Taiwan from April 1 to July 31, 2022. Children aged 2 months to 18 years, experiencing a fever of ≥ 38 °C (measured at the tympanic membrane) and seizure movements as defined by the International League Against Epilepsy (ILAE) 2017 criteria, were divided into two groups: those with confirmed acute SARS-CoV-2 infection through reverse transcription-polymerase chain reaction (RT-PCR) or rapid nasopharyngeal swab testing, and those who tested negative for the infection. Pre-existing conditions including history of epilepsy, congenital central nervous system malformations, congenital metabolic disorders, central nervous system malignancy, psychiatric and behavior disorders, history of encephalitis, developmental delay, and receiving neuropsychiatric medications are excluded. Children with non-seizure movements including chills, jitteriness, and tremors were also excluded. If a patient had multiple PED visits, only the first records were analyzed. These two groups of febrile and seizures were further categorized into two groups with encephalopathy and non-encephalopathy, based on the guidelines outlined by the Japanese Society of Child Neurology for acute encephalopathy (Fig. 1). The acute encephalopathy group was defined as impairment of consciousness of acute onset, with severity of Japan Coma Scale 20 or Glasgow Coma Scale (GCS) < 11, and with duration of 24 h or longer. The conscious impairment onset occurred during acute infection and there was evidence of brain inflammation such as brain edema in cranial computed tomography (CT) or magnetic resonance imaging (MRI) [10].

### Data collection

We collected the following information for all patients: (1) demographics, family history and past history of febrile seizures and neurological diseases; (2) symptoms and signs during illness; (3) vital signs at the initial PED encounter and discharge; (4) seizure semiology including aura, seizure pattern, associated symptoms, ictal duration, and post-ictal condition; (5) medical management at the PED; (6) laboratory and imaging results at the PED; (7) initial impression and final diagnosis at discharge from the PED, ordinary ward, or intensive care unit (ICU); and (8) neurological condition at discharge. The history of SARS-CoV-2 vaccination was obtained from Taiwan’s National Health Insurance Research Database (NHIRD). Vital signs including body temperature, respiration rate, heart rate, systolic blood pressure, and diastolic blood pressure were checked after admission to the PED. The missing data of clinical information on symptoms, signs during illness, seizure characteristics, post-ictal conditions, and neurological status at discharge were confirmed through telephone interviews with the patients and their families. Patients with incomplete data were excluded.

The non-encephalopathy group was defined as the initial presentation suggesting encephalopathy with febrile seizures, but the final diagnosis revealing another entity, including simple febrile seizures, myoclonic jerks, complex febrile seizures, and febrile seizures plus. Febrile seizures were defined as seizures occurring in children older than 1 month of age associated with febrile illness but not related to central nervous system infections. Febrile seizures were classified as either simple or complex. Simple febrile seizures were defined as a brief (less than 15 min) generalized seizure that did not recur within 24 h. Complex febrile seizures are seizures that are either prolonged (lasting longer than 15 min), focal, or recurrent within 24 h [11]. Myoclonic jerk was defined as a single or a series of brief muscle contractions, typically milliseconds in duration [12]. Febrile seizures plus was defined as febrile seizures or afebrile generalized tonic–clonic seizures that persisted beyond 5 years of age, or those with febrile seizures onset before 3 months or after 5 years of age [13, 14].

The severity of each patient was classified into three groups based on the disposition decision at the PED: (1) direct discharge from the PED was regarded as low severity; (2) admission to an ordinary ward was regarded as medium severity; and (3) admission to the ICU was regarded as critical severity. The outcomes were also classified into three groups: (1) full recovery; (2) with neurological sequelae such as cognitive or memory disturbance, headache, loss of taste or smell, and myalgia; and (3) mortality.

### Data management and statistical analysis

The mean and standard deviation were computed for continuous variables like age, vital sign and laboratory results. Meanwhile, categorical variables such as sex, febrile seizure, and vaccination history, seizure type, outcome were described using frequencies and percentages. Statistical analysis was performed using GraphPad Prism software, version 9.1.2. for Macs, GraphPad Software, San Diego, California USA. Group differences were analyzed using the chi-square test or Fisher’s exact test for categorical variables, and the Student’s t test or Mann–Whitney test for continuous variables. Associations with outcomes between patients in the encephalitis and encephalitis mimics groups were determined using univariate analysis. A *P* value less than 0.05 was considered to indicate statistical significance, and all statistical tests were two-tailed.

## Results

### Demographic data

**Fig. 2:**
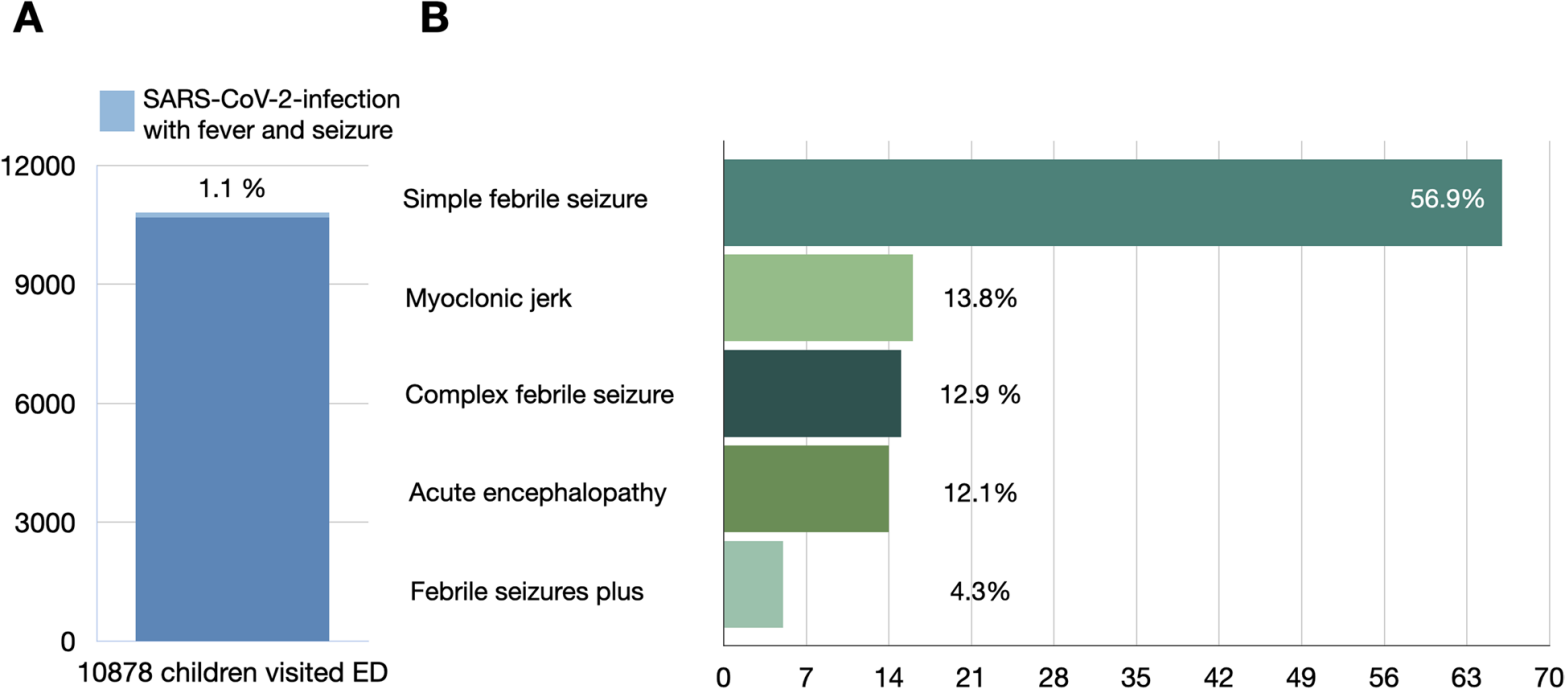
Incidence of fever with seizures in the children with COVID-19 at the pediatric emergency department. **A** After excluded 58 cases with underlying etiology and comorbidity (see exclusion criteria), the prevalence rate of the children with febrile seizures due to SARS-CoV-2 infection at the pediatric emergency department was 1.1% (116/10878). **B** The etiologies were simple febrile seizures (56.9%), myoclonic jerk (13.8%), complex febrile seizures (12.9%), febrile seizures plus (4.3%), and acute encephalopathy (12.1%)

Amidst the COVID-19 pandemic, the influx of patients to our emergency department experienced a significant surge towards the close of April 2022, concomitant with an upswing in SARSCoV-2 infections. This pattern underwent a reversal subsequent to the implementation of immunization using the Moderna and BNT vaccines for children (Supplementary Fig. 1). A total of 10,878 children (age range 2 months to 18 years) presented to our PED from April 1 to July 31, 2022, of whom 277 had symptoms of fever and seizures (Fig. 1). Among these 277 patients, 189 (68%) were diagnosed with acute SARS-CoV-2 infection by RT-PCR or rapid test from a nasopharyngeal swab. Fifteen patients were excluded from the study due to discharge against medical advice and multiple encounters, and 58 patients were excluded according to the exclusion criteria. There were 11 patients of underlying epilepsy, 2 congenital CNS malformations, 2 congenital metabolic disorders, 1 CNS malignancy, 3 psychiatric and behavior disorders, 1 past history of encephalitis, 19 seizure-mimic movement (chillness, tremor, or jitteriness), and 19 incomplete data (Fig. 1). The remaining 116 patients had suspected encephalitis, but it was difficult to make the diagnosis based on the initial symptoms. Of these patients, 14 were later diagnosed with SARS-CoV-2 acute encephalopathy, while the other 102 patients were classified into the non-encephalopathy groups (Fig. 1). Overall, 116 of the SARS-CoV-2 patients (1.1%) who presented to our PED had fever and seizures during the study period (Fig. 2A). Among these patients, 66 (56.9%) were diagnosed with simple febrile seizures, 16 (13.8%) with myoclonic jerks, 15 (12.9%) with complex febrile seizures, 5 (4.3%) with febrile seizures plus, and 14 (12.1%) with acute encephalopathy (Fig. 2B). The acute encephalopathy account 1.3 in 1000 acute SARS-CoV-2 infection children.

### COVID-19 acute encephalopathy and other COVID-19 febrile seizure

There were one acute necrotizing encephalopathy case and 13 acute encephalopathy unclassified. Except two patients presented myoclonus, others all presented generalized tonic and clonus seizure. Ten cases presented as status epilepticus and seven cases presented as cluster attack seizure. Coma, stupor, disorientation, headache, visual hallucination, slurred speech, personality change, agitation were common symptoms. Seven cases had CSF data. Except one patient with elevated protein (1829 mg/dL), and others were unremarkable. Nine patients had brain image. Severe whole brain edema, hypodensity over thalamus and brainstem on brain computed tomography, focal encephalomalacia, and lacunar infarction over bilateral lentiform nuclei had been found in three patients. Only one patient died. Five patients had neurological sequela, including intermittent auditory hallucination and agitation, headache, speech delay, slow response, and insomnia (Supplement Table 1).

**Table 1:** Clinical features and outcomes of the SARS-CoV-2 Omicron variant encephalopathy and the non-encephalopathy group

No. (%)	SARS-CoV-2 Encephalopathy (*N* = 14)	SARS-CoV-2 Non-encephalopathy (*N* = 102)	*P*
Age (year), mean ± SD	5.1 ± 3.4	2.6 ± 1.8	0.0049^*^
Sex (male) — *n* (%)	9 (64.3%)	65 (63.7%)	0.3333
Febrile seizure history— *n* (%)	3 (21.4%)	21 (20.6%)	> 0.9999
SARS-CoV-2 vaccination history – n (%)	1 (7.1%)	6 (1%)	> 0.9999
Vital sign (mean ± SD)			
Bradycardia n(%)	4 (28.6%)	0 (0%)	0.0001
Hypotension n (%)	2 (14.3%)	0 (0%)	0.0136
BT (°*C*)	39.6 ± 1.3^A^	39.5 ± 0.8^a^	0.6476
HR initial (beats/min)	129 ± 42^A^	155 ± 26^a^	0.0261^*^
RR initial (breaths/min)	22 ± 3.2^A^	25 ± 3.4^a^	0.5842
SBP initial (mmHg)	106 ± 28^B^	112 ± 19^b^	0.2311
DBP initial (mmHg)	61 ± 30^B^	75 ± 20^b^	0.0741
Systems Involvement — *n* (%)			
Respiratory	7 (50.0%)	64 (62.7%)	0.3912
Gastrointestinal	6 (42.9%)	26 (25.5)	0.2056
Cardiovascular ‡	3 (21.4%)	0 (0%)	0.0014^*^
No. of involved system	2	2	0.1328
Interval between fever and seizure (days) (mean ± SD)	1.6 ± 1.4	0.7 ± 0.9	0.0013^*^
Seizure types — *n* (%)			
GTCs	12(85.7%)	87(85.3%)	> 0.9999
Focal seizure	2 (14.3%)	2(2.0%)	0.0708
Cluster (> 1 in 24 h)	7 (50.0%)	17 (16.7%)	0.0090^*^
Status Epilepticus	7(50.0%)	14 (13.7%)	0.0037^*^
Prolong (> 15 min)	3(21.4%)	2 (2.0%)	0.0123^*^
Encephalopathy — *n* (%)			
Initial GCS (mean)	11.7	14.9	< 0.0001^*^
Conscious disturbance^§^	11 (78.6%)	3 (2.9%)	< 0.0001^*^
Disorientation	3 (21.4%)	0 (0%)	0.0014^*^
Hallucination	4 (28.6%)	0 (0%)	0.0001^*^
Acute psychosis	2 (21.4%)	0 (0%)	0.0136^*^
Initial Severity — *n* (%)			< 0.0001^*^
Discharge from ED	1 (7.1%)	66 (64.7%)	
Admission to Ward	5 (35.7%)	36 (35.3%)	
Admission to ICU	8 (57.1%)	0 (0%)	
Outcome — *n* (%)			< 0.0001^*^
Full recovered	9 (64.3%)	100 (98.0%)	
Neurological Sequelae^&^	4 (28.6%)	2 (2.0%)	
Death ††	1(7.1%)	0 (0%)	
Laboratory (mean ± SD)			
WBC (/μL)	7142 ± 3477	7848 ± 3306	0.1957
CRP (mg/L)	3.9 ± 5.3	4.3 ± 6.5	0.2808
Sugar (mg/dL)	146 ± 35.6	108 ± 24.2	0.0229^*^
pH	7.29 ± 0.11	7.39 ± 0.06	0.0018^*^
pCO_2_ (mmHg)	40.2 ± 8.9	35.9 ± 7.2	0.0780
HCO3^−^(mmHg)	19.5 ± 5.3	20.7 ± 2.2	0.9180

*Abbreviations: BT *Body temperature, *CRP *C-reactive protein, *DBP *Diastolic blood pressure, *ED *Emergency department, *GCS *Glasgow Coma Scale, *GTCs *Generalized tonic–clonic seizures, *HR *Heart rate, *ICU* Intensive care unit, *RR *Respiration rate, *SBP *Systolic blood pressure, *SD *Standard deviation, *WBC *White blood cell; Delta-vital sign (HR, RR, SBP, DBP): subtract vital sign after treatment from vital sign before treatment

^*^Statistically significance: *P* < 0.05, ‡ Cardiovascular symptoms included: bradycardia (*n* = 2), and hypotension (*n* = 3), & Neurological Sequelae: irritable (*n* = 2), headache (*n* = 1), slow response (*n* = 1) in acute encephalopathy group, poor appetite (*n* = 2) in non-encephalopathy group, †† Etiology of death: acute fulminant cerebral edema

Different cohort numbers due to missing values were represented by: ^A^*N* = 11, ^a^ *N* = 100; ^B^ *N* = 8, ^b^ *N* = 54

The diagnosis of other 102 febrile seizure cases were 66 (56.9%) simple febrile seizures, 16 (13.8%) myoclonic jerks, 15 (12.9%) complex febrile seizures, and 5 (4.3%) febrile seizures plus (Fig. 2).

#### Comparison of COVID-19 acute encephalopathy with other causes of febrile seizures

The mean age of the SARS-CoV-2 acute encephalopathy group was 5.1 ± 3.4 years old, which was significantly older than the non-encephalopathy group with mean age of 2.6 ± 1.8 years old. Both these two groups were mild male predominant with ratio of 64.3% and 63.7% in encephalopathy and non-encephalopathy group respectively. The proportion of febrile seizure history were similar between the two groups, with 21.4% and 20.6% in encephalopathy and non-encephalopathy group respectively (Table 1). The vaccination rates in both groups were low, with one (7.1%) in the acute encephalopathy group and six (1%) in the non-encephalopathy group.

#### Clinical features

The mean maximum body temperature was 39.6 ± 1.3* °C* and 39.5 ± 0.8 °C in acute encephalopathy and non-encephalopathy group respectively. The mean initial heart rate in the acute encephalopathy group was significantly lower at 129 ± 42 beats per minute compared to the non-encephalopathy group, which had a mean initial heart rate of 155 ± 26 beats per minute. Four patients (28.6%) in the acute encephalopathy group exhibited bradycardia, while two patients (14.3%) showed hypotension. Conversely, there were no occurrences of bradycardia or hypotension in the non-encephalopathy group. Multiple organ involvement was common in SARS-CoV-2 children, with a mean of two systems affected in both groups, excluding the neurological system (Table 1).

#### Neurological expressions

The seizure onset timing was significant earlier in non-encephalopathy group (mean 0.7 ± 0.9 days after fever onset) than acute encephalopathy group (mean 1.6 ± 1.4 days after fever onset). The most common seizure pattern in both groups was general tonic–clonic (GTC) seizures. In the acute encephalopathy group, cluster (70%) and status epilepticus (50%) were the second most common seizure types, showing a significant difference, and cluster attacks included repetitive GTCs or focal clonus. The longest seizure duration was 2 h in one patient from acute encephalopathy group. It presented as several times GTCs attacked without conscious recovered between the attacks and associated with bradycardia, and hypotension. The initial Glasgow Coma Scale (GCS) and level of consciousness were significantly lower in the acute encephalopathy group, with mean GCS scores of 11.7 and 14.9 in each respective group. Additionally, other forms of encephalopathy, including disorientation, hallucination, and acute psychosis, were observed (Table 1).

#### Laboratory findings and outcomes

Among the inflammatory markers, there was no leukocytosis or elevated c reactive protein in both groups. However, more severe of hyperglycemia (mean 146 ± 35.6 mg/dL) and acidosis (mean pH 7.29 ± 0.11) were presented in acute encephalopathy group with significant different from non-encephalopathy group (Table 1). The ICU hospitalization rate was significantly higher in the acute encephalopathy group (57.1%), representing a much higher initial severity. All the acute encephalopathy patients survived except for one died from acute fulminant cerebral edema. Four patients from the acute encephalopathy group presented with neurological sequela including persist irritable, headache, and slow response.

#### Summary of significant findings

There were significant differences between the two groups in terms of age, seizures occurring beyond the first febrile day, early-stage bradycardia, cluster attacks, and the presence of status epilepsy, hyperglycemia, and metabolic acidosis.

## Discussion

Seizures is a common symptom presented in 20% of hospitalized children infected with SARS-CoV-2 Omicron [15]. A study in the U.S. also found that febrile seizures occurred in 0.5% of pediatric COVID-19 patients [16]. While most seizures are transient, approximately 12% of COVID-19 patients with neurological complications experience life-threatening conditions, while acute encephalitis/encephalopathy contributes to 44% [15]. Infection-triggered encephalopathy syndrome (ITES) is an emerging concept regarding parainfection encephalitis in children, which could be utilized for diagnosing cases of COVID19 acute encephalitis / encephalopathy. The diagnosis refers to the identification of abnormal immune responses induced by viruses or bacteria, resulting in severe brain edema, necrosis, demyelination, or dysfunction. And complex multiple genetic and environmental risk factors had been suspected. Environmental risk factors include pathogens such as SARS-CoV-2, influenza, human herpesvirus-6/7, respiratory syncytial virus, and mycoplasma, as well as drugs like aspirin in Reye syndrome, non-steroidal anti-inflammatory drugs in acute necrotizing encephalopathy (ANE), and theophylline in acute encephalopathy with biphasic seizures and late reduced diffusion (AESD). Additionally, genetic risk factors are also suspected, such as RANBP2 in ANE and MYRF in mild encephalitis/encephalopathy with reversible splenial lesion (MERS) [17]. A study conducted in Japan also observed that SARS-CoV-2 appears to have a higher likelihood of causing severe acute encephalopathy compared to other viruses [14]. A review article by Hiroaki Nagase et al. indicated that immunotherapy, including corticosteroids, tocilizumab, or plasma exchange within 24 h (T1–T2) of onset, may reduce sequelae in cases of acute necrotizing encephalopathy. These findings suggest that early identification and prompt, targeted treatment in febrile seizure patients who may progress to encephalitis/encephalopathy could be beneficial [17]. Discussion of early prediction factors for acute encephalitis/encephalopathy is uncommon in articles. Kenta Kajiwara and Hiroshi Koga reported that the inability to halt seizures with a single dose of diazepam may signal a risk of acute encephalitis or bacterial meningitis [9]. Taking into account the distinct mechanisms of febrile convulsions and acute encephalitis, we examined several variables, including seizure characteristics, vital signs, and laboratory tests reflecting sympathetic tone, inflammation, and brain dysfunction. We identified some potential predicting factors.

The identified characteristics encompass age among patients, seizure beyond the first febrile day, early-stage bradycardia, cluster attacks, and the of status epilepsy, hyperglycemia and metabolic acidosis.

The mean age (95% CI) was 5.1 (3.09–7.03) years in the acute encephalopathy group and 2.6 (95% CI: 2.27–2.97) years in the non-encephalopathy febrile seizure group. Kim JM et al. reported that late-onset febrile seizures or febrile seizures plus (age above 60 months) are not uncommon, accounting for 20% (16/81) of pediatric febrile seizure patients with COVID-19 during the omicron period [18]. Therefore, age above 60 months cannot rule out febrile seizures. The mean time lapse (95% CI) between fever onset and seizure occurrence in acute encephalopathy was 1.6 (0.8–2.4) days in our studies, with the longest seizure onset day occurring up to the fifth febrile day. In the non-encephalopathy febrile seizure group, all seizure attacks occurred within the first febrile day. A previous study of 688 febrile convulsion patients also reported that the interval between fever onset and febrile seizure was within a median (IQR) of 8.0 (2.5–15) hours [9]. Therefore, seizures occurring beyond the first febrile day should raise awareness of the risk of acute encephalitis.

The postulated mechanism underlying these symptoms is believed to stem from an inflammatory/autoimmune process that unfolds throughout the disease course. Clinically, this process results in an increased frequency of cluster attacks and status epilepsy, alongside subsequent elevation in intracranial pressure, or brainstem involvement with autonomic dysfunction. This cascade finally leads to the manifestation of bradycardia and prolonged conscious disturbance. The autonomic presentation had been reported in autoimmune encephalitis, which further reinforces our findings [19]. The presence of hyperglycemia and metabolic acidosis were significantly different compared to the non-encephalopathy group, indicating stress and severity of the disease [20, 21]. Contrary to our expectations, the severity of leukocytosis, C-reactive protein levels, and highest body temperature did not indicate the risk of acute encephalitis.

Prolonged and clustered seizures were more prevalent in the acute encephalopathy group, suggesting potential differences in the mechanisms of seizure generation between encephalitis and febrile convulsions. This aligns with previous studies conducted in Japan, which have found that prolonged or refractory febrile seizures increase the risk of acute encephalitis [9]. Previous studies also discussion about the specific seizure characteristics related to autoimmune encephalitis. For instance, faciobrachial dystonic seizures (FBDS) are linked with limbic encephalitis associated with leucine-rich glioma-inactivated protein 1 (LGI1) antibodies [22]. Seizures often present as a prominent symptom in cases of LGI1 and glutamic acid decarboxylase (GAD) antibody encephalitis, while they manifest as a late-stage symptom in cases of N-methyl-D-aspartate receptor (NMDAR) antibody encephalitis. Compared to typical mesial temporal lobe epilepsy (MTLE) with hippocampal sclerosis (HS), autoimmune encephalitis tends to exhibit a shorter ictal duration, a higher frequency of daily focal impaired awareness seizure, and a lower incidence of postictal confusion [23]. In our study, we observed that GTCs were the prevailing seizure type, both in cases of COVID-19 acute encephalopathy and in the non-encephalopathy group. Despite this, no statistically significant difference was identified between the two groups in this regard.

Myoclonus has been linked to SARS-CoV-2 infection as documented in prior research [24]. In our investigation, we identified a total of 16 cases featuring myoclonus; however, none of these cases progressed into acute encephalopathy or encephalitis. This stands in contrast to cases of rhombencephalitis associated with enterovirus infection, where myoclonus has been identified as an early and autonomous indicator of more severe disease progression [25].

We performed the study try to improve the clinical management of febrile seizure in acute phase. Recognizing these risk factors can aid healthcare providers in early identification, prompt intervention, and resource allocation, ultimately enhancing the management of pediatric COVID-19 cases with acute encephalopathy. However, there were limitations need to mention in applying our findings. This is a retrospective study with small sample size of population from PED and short study period, which may have led to an underestimation of the entire affected population, particularly those with milder disease may not have presented to our PED. Except one acute necrotizing encephalopathy, other COVID19 acute encephalopathy were classified in acute encephalopathy unclassified. All of the acute encephalopathy unclassified have good prognosis. Thus, applying our finding to other encephalitis such as fulminant cerebral edema encephalitis, acute disseminated encephalomyelitis, antibody positive autoimmune encephalitis such as limbic encephalitis or anti-NMDAR encephalitis should be careful. Some missing data of CSF analysis and brain image in our study might also lead to the precision diagnosis in the acute encephalopathy group. Further prospective cohort studies and case–control studies, encompassing other types of COVID-19 encephalopathy, are needed to examine our findings and explore their applicability.

## Conclusion

Seizures occurring beyond the first day of fever, bradycardia, clustered seizures, status epilepticus, hyperglycemia, and metabolic acidosis in pediatric patients with COVID-19-related febrile seizures should raise concerns about acute encephalitis/encephalopathy. The highest body temperature and the severity of leukocytosis or C-reactive protein levels were not linked to poor outcomes.

### Written informed consents

I understand that the text and any pictures published in the article will be freely available on the internet and may be seen by the general public. The pictures and text may also appear on other websites or in print, may be translated into other languages or used for commercial purposes. I have been offered the opportunity to read the manuscript.

### Supplementary Information

**Additional file 1: Table S1.** Summary of clinical characters of 14 patients with COVID-19 encephalopathy.

**Additional file 2: Figure S1.** Pediatric COVID-19 Cases during Omicron Epidemic at Chang Gung Memorial Hospital, April-July 2022. Percentage of patients with COVID-19 in pediatric emergency department each day during the epidemic of Omicron variant from April to July, 2022 in Chang Gung Memorial Hospital. COVID-19: coronavirus disease 2019; PER: pediatric emergency room. Moderna: Moderna mRNA COVID-19 vaccine; BNT162b2: Pfizer-BioNTech 162b2 mRNA COVID-19 vaccine.

## Data Availability

The datasets used and/or analyzed during the current study are available from the corresponding author on reasonable request.

## Abbreviations

CI: Confidence interval
CT: Computed tomography
FBDS: Faciobrachial dystonic seizures
GAD: Glutamic acid decarboxylase
GCS: Glasgow Coma Scale
GTCs: General tonic–clonic
HS: Hippocampal sclerosis
ICU: Intensive care unit
ILAE: International League Against Epilepsy
LGI1: Leucine-rich glioma-inactivated protein 1
MRI: Magnetic resonance imaging
MTLE: Mesial temporal lobe epilepsy
NHIRD: National Health Insurance Research Database
NMDAR: N-methyl-D-aspartate receptor
PED: Pediatric emergency department
RT-PCR: Reverse transcription-polymerase chain reaction
