# Supplementary material for: Predicting factors for acute encephalopathy in febrile seizure children with SARS-CoV-2 omicron variant: a retrospective study
Source: BMC Pediatr. 2024 Mar 25;24:211. doi: 10.1186/s12887-024-04699-x (PMC10962131; doi:10.1186/s12887-024-04699-x)
Supplement: Supplementary file 1 — Additional file 1: Table S1. Summary of clinical characters of 14 patients with COVID-19 encephalopathy. [file 12887_2024_4699_MOESM1_ESM.docx]

Supplementary Table 1 Summary of clinical characters of 14 patients with COVID-19 encephalopathy.

| Pt | Age& Gender |  | Seizure | | | Symptoms | CSF | EEG | Brain MRI | Neurological sequela |
| --- | --- | --- | --- | --- | --- | --- | --- | --- | --- | --- |
|  |  | Final Diagnosis | Pattern | Duration | Cluster |  |  |  |  |  |
| 1 | 3.2 M | acute necrotizing encephalopathy | Generalized | RSE | Y | Coma  chillness, acrocyanosis, refractory hypotension | WBC 3/uL  RBC 12/uL  Protein 1829mg/dL | - | Severe brain edema with central herniation Hypodense over bilateral thalamus and brainstem (CT) | Death |
| 2 | 5.6 M | acute encephalopathy unclassified | Generalized and Focal | SE | Y | Conscious disturbance, hallucination  Cough, rhinorrhea, vomiting, headache, lethargy, | WBC 0/uL RBC 0/uL Protein  19.1 mg/dL | DBS  Focal ED | Negative | Intermittent auditory hallucination and agitation |
| 3 | 10.9 M | acute encephalopathy unclassified | Generalized | SE | N | Stupor, severe headache, dizziness, vomiting, ataxia | WBC 1/uL  RBC580/uL  Protein 19 mg/dL | DBS  No ED | Negative | No |
| 4 | 12 M | acute encephalopathy unclassified | Generalized | SE | Y | Slurred speech, drooling, lethargy, headache, personality change | WBC 15 RBC 46000 Protein 45.7 mg/dL | - | - | No |
| 5 | 7.3 M | acute encephalopathy unclassified | Generalized and Focal | SE | N | dystonia, hallucination, acute psychosis, disorientation, hypotension, and bradycardia | - | DBS  Focal ED | Negative | Chronic headache |
| 6 | 0.9 F | acute encephalopathy unclassified | Generalized | SE | Y | Stupor, excessive daytime sleep, cough, rhinorrhea, sore throat, bradycardia | - | - | Negative | No |
| 7 | 1.2 F | acute encephalopathy unclassified | Generalized | SE | Y | Coma, bradycardia  Cough, rhinorrhea, sore throat | WBC 0/uL  RBC 1/uL  Protein 16.7mg/dL | DBS  Focal ED | Negative | Speech delay |
| 8 | 3.2 F | acute encephalopathy unclassified | Generalized | 2mins | Y | Hallucination, Conscious disturbance, | WBC 0/uL  RBC 1/uL  Protein 21.8/uL | Focal ED | Negative | No |
| 9 | 6.4 M | acute encephalopathy unclassified | Generalized | SE | Y | Conscious disturbance, excessive daytime sleep | WBC 0/uL  RBC 1/uL  Protein 29.7/uL | Focal ED | Negative | No |
| 10 | 3.2 M | acute encephalopathy unclassified | Myoclonus | SE | N | Coma, visual hallucination, bradycardia, hypotension | - | DBS | focal encephalomalacia | Slow response for two weeks, improved subsequently |
| 11 | 4.9 M | acute encephalopathy unclassified | Generalized | 3mins | N | Agitation, Visual hallucination, personality change, bizzare and degenerative behavior,  Cough, rhinorrhea, sore throat, vomiting | - | Focal slow wave | lacunar infarction over bilateral lentiform nuclei | Insomnia |
| 12 | 6.5 F | acute encephalopathy unclassified | Myoclonus | SE | N | Conscious disturbance Cough, rhinorrhea, sore throat, hypotension | - | - | Negative | No |
| 13 | 0.9 M | acute encephalopathy unclassified | Generalized | SE | N | Conscious disturbance, vomiting,  Cough, rhinorrhea, sore throat, | - | - | - | No |
| 14 | 4.2 M | acute encephalopathy unclassified | Generalized | SE | N | Conscious disturbance  Cough, rhinorrhea, | - | - | - | No |

Abbreviations: M: male, F: female, Temp: temperature, RSE: refractory status epilepticus, SE: Status epilepticus; EEG: electroencephalogram; CT: computer tomography; MRI: magnetic resonance imaging; DBS: diffuse background slow; ED: epileptiform discharge
