# Supplementary figures and images for: Predicting factors for acute encephalopathy in febrile seizure children with SARS-CoV-2 omicron variant: a retrospective study
Source: BMC Pediatr. 2024 Mar 25;24:211. doi: 10.1186/s12887-024-04699-x (PMC10962131; doi:10.1186/s12887-024-04699-x)

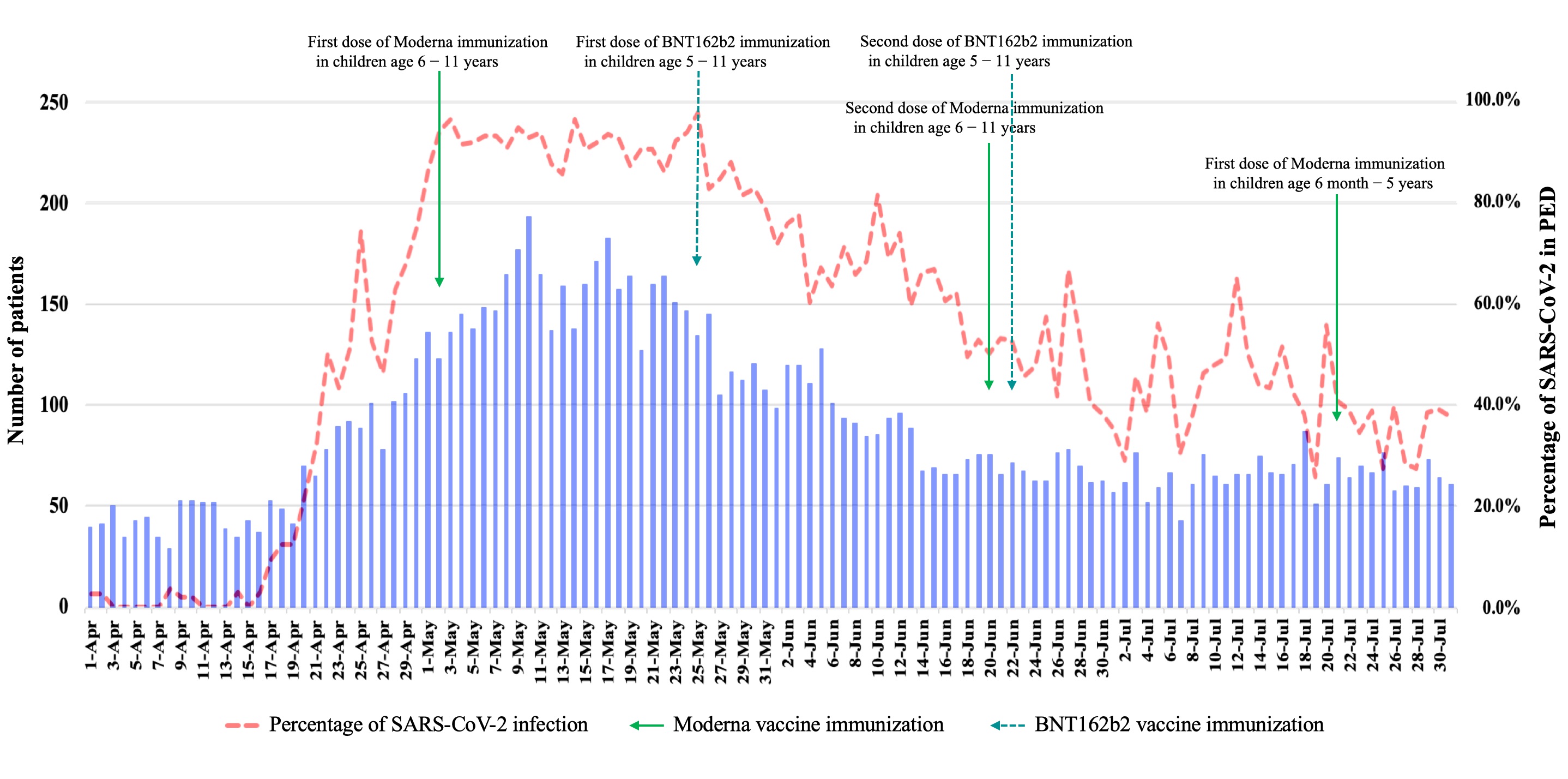

Supplement: Supplementary file 2 — Additional file 2: Figure S1. Pediatric COVID-19 Cases during Omicron Epidemic at Chang Gung Memorial Hospital, April-July 2022. Percentage of patients with COVID-19 in pediatric emergency department each day during the epidemic of Omicron variant from April to July, 2022 in Chang Gung Memorial Hospital. COVID-19: coronavirus disease 2019; PER: pediatric emergency room. Moderna: Moderna mRNA COVID-19 vaccine; BNT162b2: Pfizer-BioNTech 162b2 mRNA COVID-19 vaccine. [file 12887_2024_4699_MOESM2_ESM.jpeg]
